# Approaches to Research in Art Therapy Using Imaging Technologies

**DOI:** 10.3389/fnhum.2019.00159

**Published:** 2019-05-17

**Authors:** Juliet L. King, Girija Kaimal

**Affiliations:** ^1^Department of Art Therapy, The George Washington University, Washington, DC, United States; ^2^Department of Neurology, School of Medicine, Indiana University, Indianapolis, IN, United States; ^3^Department of Creative Arts Therapies, Drexel University, Philadelphia, PA, United States

**Keywords:** art therapy, neuroscience, mobile brain/body imaging, fNIRS (functional near infrared spectroscopy), EEG

“*There is no insurmountable solitude. All paths lead to the same goal: to convey to others what we are*”

- *Pablo Neruda*

Artistic expression is a part of being human and there are many theories about its role and purpose in our lives. In the profession of art therapy, art making is an integral part of treatment and takes place in the context of a facilitative/therapeutic relationship with a trained art therapist. Art therapists use a range of artistic media and intervention strategies to engage the client in creative expression with the aim of symptom reduction. Although the profession has produced substantial scholarship documenting its clinical value, identifying the mechanisms of change through art therapy has been a challenge due to the multiple psychic processes that are involved in psychotherapeutic care, made even more complex with the additional components of artistic expression. Contemporary neuroimaging technology offers an accessible method of measuring brain dynamics in real world environments. This technology could help to elucidate how art making within the therapeutic relationship engages the brain and provide scientific data to measure mechanisms of change and outcomes of clinical art therapy interventions.

## Overview of Art Therapy

Artistic expression has accompanied human evolution and several theories have been proposed about it role and purpose in our lives (Dutton, 2009; Winner, 2018). These include its identified roles in evoking emotions and imagination, stimulating cognition, and, enabling communication. In the field of art therapy, art making occurs in the context of a facilitative/therapeutic relationship with a trained art therapist and is considered reparative and adaptive because it taps into the creative and imaginative aspects of human brain functioning. The American Art Therapy Association (2019) defines art therapy as an integrative mental health and human services profession that enriches the lives of individuals, families, and communities through active art making, creative process/theory, applied psychological theory, and human development within a psychotherapeutic relationship. Art therapy originated in the twentieth century simultaneously in many parts of Europe and America in response to the needs of clinical populations who were not being served effectively with traditional approaches to mental and physical health. Over the past several decades, art therapists have gone on to work with a range of populations including those facing developmental challenges and impacts of adverse events (Tripp, as cited in King, 2016), in part due to the value of creative, symbolic communication in the reconsolidation of traumatic memories and emotional regulation. Although art therapy is increasingly well-known and established as a clinical services profession, research in the field is needed to better understand the mechanisms of change and how art therapy assessments and interventions impact patient/client outcomes.

## Neuroimaging Research in Art Therapy

Art therapy is a profession grounded in assumptions of neuroplasticity and sensorimotor engagement and therefore aligns well with research protocols in neuroscience. It is however, a challenge to quantify subjective aspects of an individual's creative process and this is one of the reasons why it has been difficult to test art therapy interventions with the rigor and generalizability necessary to generate evidence on the effectiveness of an intervention. Creativity from a neuroscientific perspective is considered a compound construct in and of itself (Dietrich, 2019). Art therapy relies heavily on creative, symbolic self-expression as a method of engaging and objectifying less conscious cognition, emotion and memories, and in this way makes it easier for a person to “see” and eventually put words to psychic processes that are not otherwise readily attainable. In other words, art therapy can be a way of telling without talking (Cohen and Cox, 1995). Understanding and identifying the brain processes involved in this type of communication will help to explain with more certainty why and how art therapy works.

In an attempt to conceptualize the theoretical underpinnings of neuroscience and art therapy and provide a framework to propel the systematic integration of the two fields, King (2016) developed three initial tenets within which to consider art therapy as neurotherapeutic. Although theoretical at this point, these tenets propose mechanisms of change through art therapy based on observations of clinical sessions: (1) The art-making process and the artwork itself are integral components of treatment that help to understand and elicit verbal and non-verbal communication within an attuned therapeutic relationship; (2) Creative expression is healing and life enhancing; and; (3) The materials and methods utilized affect self-expression, assist in emotional self-regulation, and are applied in specialized ways. These tenets help to distill and organize the multiple components of art therapy and provide a foundation from which to systematically research processes and outcomes. A key consideration in assessing brain function in the context of art therapy is the ability to track human functioning in natural ways, as action is involved in art making and movement is an integral part of the process. Although the mechanisms in our brain do not often map very well with what we experience (King, 2018), the introduction of Mobile Brain/Body Imaging (MoBI) (Makeig et al., 2009) to the range of contemporary imaging tools has increased these capacities.

Art making and interpersonal interactions in an art therapy session are ideally suited for study using MoBI technologies in that they align with measurements in real world environments. For example, electroencephalography (EEG) and functional near infrared spectroscopy (fNIRS) are non-invasive tools that monitor participants brain activity while allowing for free movement and interpersonal interactions in the rest of the body. EEG measures brain wave activity while fNIRS is a hemodynamic activity-based neuroimaging technique that measures the relative changes in concentration of oxygenated and deoxygenated hemoglobin secondary to neuronal activity (Scholkmann et al., 2014). These MoBI set-ups allow a person to actively engage in the art-making process and to work with a range of art materials without being restricted by the technological equipment. Aligning scientific measurement with a natural therapeutic setting should enable the generation of findings that are relevant to clinical contexts.

The use of EEG to understand brain processes connected to artistic self-expression is in the initial stages of exploratory research. King et al. (2017) found that art making resulted in overall increased power as measured by EEG compared with a rote motor task, highlighting the fact that there are differences in brain activation in a creative vs. a pure sensorimotor-based activity. This study provided initial support for the use of MoBI to assess the interaction of movement, cognition, and brain dynamics. Art therapy researchers have used EEG and fNIRS as imaging tools in a few pilot studies. Using quantitative electroencephalography (qEEG), Belkofer et al. (2014) investigated the differences in patterns of brain activity among artists and non-artists during the process of drawing. Results indicated that there was more activity in the left hemisphere of the brains of artists, whereas more activity was reflected in the frontal lobe of non-artists. This result may have been based on the fact that drawing was a new task for them and that stimulation in this area of the brain is a sign of learning. There was an increased presence of alpha waves for both the artists and the non-artists, indicating the potential for relaxation through creative opportunities generated by drawing tasks. Similarly, in a qEEG comparison of working with clay and drawing, activation was noted in regions of memory processes, meditative states, and spatiotemporal processing (Kruk et al., 2014). Kaimal et al. (2017a) examined the outcomes of three different drawing tasks on reward perception as measured using fNIRS. The underlying assumption in this study was that blood flow in the medial prefrontal cortex would indicate activation of one of the reward pathways in the brain. Participants were given three drawing tasks (coloring, doodling, and free drawing) spanning 3 min, each with intermittent rest periods of 2 min each. The findings indicated that all the drawing tasks activated this reward pathway of the brain compared with the no-activity rest conditions, with the doodling condition resulting in maximum activation.

These studies highlight preliminary work in identifying mechanisms of change in art making that require further research: Art making within the therapeutic relationship elicits creativity, evokes positive emotional states of relaxation, and is influenced by specific differences in art media, art task and perceived skills. These are areas to explore further and might be organized according to the aforementioned tenets of art therapy (King, 2016). Doing so would help to clarify methods of systematically integrating art therapy and neuroscience theory, practice and research.

## Potential Approaches to Research in Art Therapy Using Mobi

A challenge in art therapy and neuroimaging research lies in linking relevant brain functions with the appropriate psychological and behavioral constructs. The impact of the psychotherapeutic relationship is difficult to prove whether one practices traditional (verbal) psychotherapy or complementary therapies such as art and expressive therapies (King, 2016). The nature and quality of the therapeutic interaction is difficult to study in any psychotherapeutic approach, and is exacerbated with the ambiguity and subjectivity that accompany artistic processes and products. Despite these challenges, in our opinion there are some components of art therapy practice that can be studied systematically using neuroimaging modalities including tracking the patient/client's experience, responses to viewing artwork, the experience of the art making process, and, the interactions with the art therapist. For example, researchers might examine whether mental imagery experienced and eventually externalized through the creative process in art therapy treatment results in functional changes in the brain. These changes might be observed in frequency, time, and connectivity using EEG. Functional connectivity of cortical brain networks can be identified and measured with the use of EEG and contemporary neuroimaging. By investigating a shift in cortical brainwave activity throughout an art therapy intervention, it may be possible to measure a change in alpha wave activity that provides data about the deeper brain networks responsible for memory recall. Utilizing an assessment such as PTSD checklist pre and post art therapy treatment and then comparing the results of this assessment with a change in alpha wave activity would help to distill the correlations between brain function and creative expression and allow us to narrow the identified networks involved with traumatic memory recall. Another approach could include tracking the role of visual perception and activation of the default mode network (DMN) since it is increasingly being recognized as being relevant to self-reflection (Immordino-Yang et al., 2012), imaginal cognition, and aesthetic experiences (Belfi et al., 2019). EEGs of artists vs. non-artists engaging in artmaking might enable tracking activation of alpha waves associated with DMN activation, relaxation, meditation, and daydreaming. Stevens and Zabelina recently (2019) identified alpha, theta and gamma wave patterns as markers of creativity. Examination of creativity and its interaction with art media within a specific population is a mechanism of change that can be examined using EEG. One of the recurring outcomes of arts engagement is the evocation of positive emotions (Collie et al., 2006; Kaimal and Ray, 2017; Kaimal et al., 2017b). Potential areas of inquiry using fNIRS could include examining positive emotions and reward perception through the dopaminergic pathways in the medial prefrontal cortex and the parietal cortex (Lambert, 2010; Hass-Cohen and Findlay, 2015). fNIRS also captures overall prefrontal cortex activation which can be used as a measure of executive function and self-regulation for disorders of addiction (Bunce et al., 2013), suicidality (Sargent et al., 2018), depression (Ma et al., 2017) and binge eating (Nasser et al., 2016). The role that the facilitated use of art media in art therapy sessions plays in executive control and emotion regulation can be examined further by tracking PFC activation using fNIRS.

MoBI technologies also have the capacity to propel the testing of aspects of the therapeutic relationship. For instance, Dikker et al. (2017) described an advanced understanding of brain processes in a social context and found that brain-to-brain synchrony is a possible neural marker for dynamic social engagement that is likely driven by shared mechanisms of attention (King, 2019). The relational component, the synchrony between the therapist and the patient/client, and the functional and structural changes that occur in the patient/client's brain are still poorly understood and EEG and fNIRS provide the opportunity to explore and examine what is happening between people as they relate to one another in interactive environments. Most studies to date have focused on relatively healthy populations but these tools are particularly relevant in research involving the treatment of psychological trauma. It is now common knowledge that central nervous system and neurobiological mechanisms of stress response and memory recall are compromised as a result of traumatic experiences and that the creative process offers an opportunity to rework and repair fragmented memories within an attuned therapeutic relationship (Walker et al., 2016, 2018; King, 2019). It is possible to consider that learning new sensory-motor associations when experiencing the same sensory input and associating these with a different output can help overwrite traumatic memories, which can be measured and tested with this innovative technology (King, 2018). The occurrence of alpha wave attenuation in individuals with PTSD could be examined using brain synchronization in group work including attunement, exploration of the mirror neuron system in relational therapeutic exchange, and the potential connections to empathy. Utilizing these modalities can expand the knowledge base related to the observation that movement directly impacts emotional regulation (Shafir, 2016). Thus, there might be opportunities to investigate the complicated aspects of psychotherapeutic assessment and intervention including eye and head movements that occur during the creative process. In some cases, virtual reality technology offers avenues for embodied artistic expression which could be different from outcomes and processes seen in traditional artistic media (Kaimal et al., 2019).

Lastly, although not mobile, positron emission tomography and functional magnetic resonance imaging studies could examine structural as well as functional outcomes of art therapy. For example, Walker et al. (2018) found correlations between visual themes of social connectedness and improved thalamic functioning in patients diagnosed with post-traumatic stress. Changes in amygdala and hippocampal volume might be other structural changes to track in combination with functional brainwave shifts seen during art making assessed through MoBI. Future interdisciplinary research studies could examine the links between key behavioral and psychosocial changes (as seen from clinical practice by art therapists) including creative capacity, improved mood and emotion regulation with associated changes in brain functioning (identified by and imaging experts).

## Conclusions

Art therapy sessions offer a complex and multifaceted interaction between the patient/client and the therapist. The sessions involve art making and depends on the process and the artwork to facilitate adaptive change. MoBI technology can offer new insights into the mechanisms of change through art therapy including examining assessment and treatment outcomes in naturalistic settings. As these tools develop further, we will be able to better capture multiple dimensions of the lived experience of the patient/client and therapist including simultaneous physiological and psychological shifts. This paper seeks to invite interdisciplinary collaborations that include imaging specialists, engineers, neurologists, neuroscientists, psychologists, and art therapy clinicians, in order to address the question of how art therapy interactions result in transformative change in adaptive human functioning.

## Author Contributions

JK and GK wrote this submission jointly. JK focused on the EEG sections and GK on the fNIRS sections based on areas of interest and previous experience working with these technologies. The remaining narrative was a collective effort.

### Conflict of Interest Statement

The authors declare that the research was conducted in the absence of any commercial or financial relationships that could be construed as a potential conflict of interest.

## References

[B1] American Art Therapy Association (2019). [Organization Web Site]. Retrieved from: https://arttherapy.org/ (accessed January 10, 2019).

[B2] Belfi A. M. Vessel E. A. Brielmann A. Isik A. I. Chatterjee A. Leder H.. (2019). Dynamics of aesthetic experience are reflected in the default-mode network. NeuroImage 188, 584–597. 10.1016/j.neuroimage.2018.12.017 30543845 PMC8493917

[B3] Belkofer C. M. Van Hecke A. V. Konopka L. M. (2014). Effects of drawing on alpha activity: a quantitative EEG study with implications for art therapy. Art Therapy 31, 61–68. 10.1080/07421656.2014.903821

[B4] Bunce S. C. Harris J. Izzetoglu K. Ayaz H. Izzetoglu M. Pourrezaei K. (2013). Functional near-infrared spectroscopy in addiction treatment: preliminary evidence as a biomarker of treatment response, in International Conference on Augmented Cognition (Berlin, Heidelberg: Springer), 250–258.

[B5] Cohen B. Cox C. (1995). Telling Without Talking: Art as a Window Into the World of Multiple Personality. New York, NY: WW Norton and Co.

[B6] Collie K. Spiegel D. Malchiodi C. Backos A. (2006). Art therapy for combat-related PTSD: recommendations for research and practice. Art Therapy 23, 157–164. 10.1080/07421656.2006.10129335

[B7] Dietrich A. (2019). Where in the brain is creativity: a brief account of a wild-goose chase. Curr. Opin. Behav. Sci. 27, 36–39. 10.1016/j.cobeha.2018.09.001

[B8] Dikker S. Wan L. Davidesco I. Kaggen L. Oostrik M. McClintock J.. (2017). Brain-to-brain synchrony tracks real-world dynamic group interactions in the classroom. Curr. Biol. 27, 1375–1380. 10.1016/j.cub.2017.04.002 28457867

[B9] Dutton D. (2009). The Art Instinct: Beauty, Pleasure, and Human Evolution. New York, NY: Bloomsbury Press.

[B10] Hass-Cohen N. Findlay J. C. (2015). Art Therapy and the Neuroscience of Relationships, Creativity, and Resiliency: Skills and Practices. New York, NY: W.W. Norton and Company.

[B11] Immordino-Yang M. H. Christodoulou J. A. Singh V. (2012). Rest is not idleness: implications of the brain's default mode for human development and education. Perspect. Psychol. Sci. 7, 352–364. 10.1177/1745691612447308 26168472

[B12] Kaimal G. Ayaz H. Herres J. M. Makwana B. Dieterich-Hartwell R. M. Kaiser D. H. (2017a). Functional near-infrared spectroscopy assessment of reward perception based on visual self-expression: coloring, doodling, and free drawing. Arts Psychother. 55, 85–92. 10.1016/j.aip.2017.05.004

[B13] Kaimal G. Carroll-Haskins K. Berberian M. Dougherty A. Carlton N. R. Ramakrishnan A. (2019). Virtual Reality in Art Therapy. Philadelphia, PA: Journal of the American Art Therapy Association.

[B14] Kaimal G. Mensinger J. L. Drass J. M. Dieterich-Hartwell R. M. (2017b). Art therapist-facilitated open studio versus coloring: differences in outcomes of affect, stress, creative agency, and self-efficacy. Can. Art Therapy Assoc. J. 30, 56–68. 10.1080/08322473.2017.1375827

[B15] Kaimal G. Ray K. (2017). Free art-making in an art therapy open studio: changes in affect and self-efficacy. Arts Health, 9, 154–166. 10.1080/17533015.2016.1217248

[B16] King J. L. (Ed.). (2016). Art Therapy, Trauma, and Neuroscience: Theoretical and Practical Perspectives. New York, NY: Routledge.

[B17] King J. L. (2018). Report from 21st century great conversations in neuroscience, art and related therapeutics. Front. Psychol. 9:1428 10.3389/fpsyg.2018.01428 30150955 PMC6099956

[B18] King J. L. (2019). “Neurobiological mechanisms underlying co-leadership,” in eds Wise Nash, *Co-Leading in Trauma Group Settings: The Art of Attunement* (New York, NY: Routledge).

[B19] King J. L. Knapp K. E. Shaikh A. Li F. Sabau D. Pascuzzi R. M. (2017). Cortical activity changes after art making and rote motor movement as measured by EEG: a preliminary study. Biomed. J. Sci. Techn. Res. 1, 1–21. 10.26717/BJSTR.2017.01.000366

[B20] Kruk K. A. Aravich P. F. Deaver S. P. deBeus R. (2014). Comparison of brain activity during drawing and clay sculpting: a preliminary qEEG study. Art Therapy 31, 52–60. 10.1080/07421656.2014.903826

[B21] Lambert K. (2010). Lifting Depression: A Neuroscientist's Hands-on Approach to Activating Your Brain's Healing Power. New York, NY: Basic Books.

[B22] Ma X. Y. Wang Y. J. Xu B. Feng K. Sun G. X. Zhang X. Q.. (2017). Near-infrared spectroscopy reveals abnormal hemodynamics in the left dorsolateral prefrontal cortex of menopausal depression patients. Disease Markers 2017:1695930. 10.1155/2017/1695930 28293062 PMC5331417

[B23] Makeig S. Gramann K. Jung T. P. Sejnowski T. J. Poizner H. (2009). Linking brain, mind and behavior. Int. J. Psychophysiol. 73, 95–100. 10.1016/j.ijpsycho.2008.11.008 19414039 PMC2796545

[B24] Nasser J. A. Ayaz H. Golen R. P. Makwana B. Albajri E. Price M. B. (2016). Changes in Neural Activity of the Prefrontal Cortex During Eating in Humans. Teddington, London: Paper presented at the meeting of British Feeding and Drinking Group, April 7-8, 2016.

[B25] Sargent A. Fedorowich C. Diamond G. Ayaz H. (2018). Neural “Correlates of Adolescent Depression and Suicide: an fNIRS Study,” Frontiers in Human Neuroscience Conference Abstract: 2nd International Neuroergonomics Conference Proceedings (Philadelphia, PA). 10.3389/conf.fnhum.2018.227.00078

[B26] Scholkmann F. Kleiser S. Metz A. J. Zimmermann R. Pavia J. Wolf U.. (2014). A review on continuous wave functional near-infrared spectroscopy and imaging instrumentation and methodology. Neuroimage 85, 6–27. 10.1016/j.neuroimage.2013.05.004 23684868

[B27] Shafir T. (2016). Using movement to regulate emotion: neurophysiological findings and their application in psychotherapy. Front. Psychol. 7:1451. 10.3389/fpsyg.2016.01451 27721801 PMC5033979

[B28] Stevens C. E. Jr Zabelina D. L. (2019). Creativity comes in waves: an EEG-focused exploration of the creative brain. Curr. Opin. Behav. Sci. 27, 154–162. 10.1016/j.cobeha.2019.02.003

[B29] Walker M. Kaimal G. Koffman R. DeGraba T. J. (2016). Art therapy for PTSD and TBI: a senior active duty military service member's therapeutic journey. Arts Psychotherapy 49, 10–16. 10.1016/j.aip.2016.05.015

[B30] Walker M. S. Stamper A. M. Nathan D. E. Riedy G. (2018). Art therapy and underlying fMRI brain patterns in military TBI: a case series. Int. J. Art Therapy 23, 180–187. 10.1080/17454832.2018.1473453

[B31] Winner E. (2018). How Art Works: A Psychological Exploration. New York, NY: Oxford University Press.

